# Rosuvastatin Induces Renal HO-1 Activity and Expression Levels as a Main Protective Mechanism against STZ-Induced Diabetic Nephropathy

**DOI:** 10.3390/medicina58030425

**Published:** 2022-03-15

**Authors:** Gehan H. Heeba, Marwa A. M. Ali, Azza A. K. El-Sheikh

**Affiliations:** 1 Pharmacology and Toxicology Department, Faculty of Pharmacy, Minia University, Minia 61511, Egypt; ghhh70@yahoo.com; 2 Abu Qurqas Health Administration, Minia Directorate of Health, Ministry of Health, Minia 61611, Egypt; marwa_g88@yahoo.com; 3 Basic Health Sciences Department, College of Medicine, Princess Nourah Bint Abdulrahman University, P.O. Box 84428, Riyadh 11671, Saudi Arabia

**Keywords:** diabetic nephropathy, heme oxygenase-1, myeloperoxidase, rosuvastatin, TNF-α

## Abstract

*Background and Objectives:* Nephroprotective effect of statins is still controversial. The aim of this study was to investigate the possible hemin-like nephroprotective effect of rosuvastatin (RSV) in streptozotocin (STZ)-induced diabetic rats. *Materials and Methods:* DN was induced in rats via a single dose of 50 mg/kg STZ i.p., with or without RSV (10 mg/kg orally) for 30 days. To investigate hemin-like effect of RSV on renal heme oxygenase-1 (HO-1), RSV was administered in the presence or absence of an inhibitor of HO-1; zinc protoporphyrin-XI (ZnPP), in a dose of 50 µmol/kg i.p. *Results:* Induction of diabetes with STZ caused, as expected, significant hyperglycemia, as well as deteriorated kidney function, lipid profile and histopathological architecture. The DN group also showed renal oxidative stress, indicated by decreased superoxide dismutase, catalase, and reduced glutathione, with increased malondialdehyde, myeloperoxidase and nitric oxide. Renal expression of inflammatory marker TNF-α, and pro-apoptotic marker caspase 3, were also increased in the DN group. Administration of RSV in DN rats did not improve glucose level but succeeded in recovering kidney function and normal structure as well as improving the lipid profile. RSV also improved renal oxidative, inflammatory, and apoptotic statuses. Interestingly, the administration of RSV increased renal expression and activity of HO-1 compared to the untreated DN group. Co-administration of ZnPP blocked the effect of RSV on HO-1 and deteriorated all RSV favorable effects. *Conclusions:* RSV can protect against DN, at least in part, via increasing renal HO-1 expression and/or activity, which seems to be upstream to RSV antioxidant, anti-inflammatory, and anti-apoptotic effects.

## 1. Introduction

Diabetic nephropathy (DN) is one of the main reasons for the high global incidence of end-stage renal disease [1]. This is mainly due to the increased prevalence of obesity-associated diabetes mellitus, with nearly half of diabetic patients eventually developing DN [2]. Despite that the pathophysiology of development and progression of DN has been thoroughly investigated. However, the mechanisms involved are not yet completely elucidated. One of the major pathways incriminated in launching a cascade of events leading to the development of DN is the oxidative stress pathway, where renal mitochondria produce reactive oxygen species (ROS) as a response to chronic hyperglycemia, which may initiate renal hypoxia and, in turn, DN [3]. Either ROS or the chronic hyperglycemia itself can further trigger diverse signaling mediators, including inflammatory cytokines as tumor necrosis factor (TNF)-α, chemokines, transcription factors and vasoactive compounds [4], which may elicit apoptosis causing permanent functional and structural renal damage. Still, the oxidative, inflammatory, and apoptotic pathways intermingle with high complexity making it difficult to highlight a distinct cause–effect relationship between them [5]. Recently, the heme oxygenase pathway has also been implicated to have a role in DN by reducing podocyte autophagy and diminishing apoptosis [6]. Traditionally, the initial diagnostic sign of DN is albuminuria, with urinary albumin of 30–299 mg/g creatinine indicative of early nephropathy, and more than 300 mg/g creatinine signifying overt nephropathy [7]. A urinary level of heme oxygenase-1 (HO-1) has also been detected in diabetic patients, even prior to the detection of any apparent albuminuria, and was suggested as an early biomarker for DN [8]. We have previously shown the crucial role of HO-1 as one of the mechanisms involved in the nephroprotection in diabetic rats, where we succeeded to alter the progress of DN using hemin and zinc protoporphyrin-IX (ZnPP); an inducer and an inhibitor of HO-1 enzymatic activity, respectively [9]. 

The association between a decreased risk of development of DN in patients and an increased serum level of high density lipoprotein-C (HDL-C) has also been suggested [10], indicating a possible role of anti-dyslipidemic drugs in the prevention and/or treatment of DN. Rosuvastatin (RSV) is a synthetic statin that decreases endogenous cholesterol synthesis via competitive inhibition of the hepatic 3-hydroxy-3-methylglutanyl coenzyme A (HMG-CoA) reductase enzyme, inhibiting mevalonate synthesis, which is a crucial step in cholesterol biosynthesis, with higher efficacy on improving lipid profile compared to other compounds of the same category [11]. In addition to its lipid lowering effects, RSV has pleiotropic effects, probably through its reported antioxidant, anti-inflammatory, and antithrombotic properties [11,12], which made RSV one of the drugs that can beneficially contribute to prevention and/or treatment of cardiovascular diseases. However, the use of statins in general to treat DN is still controversial [13]. Here, we hypothesized that RSV had a hemin-like effect and might confer protection against DN through increasing HO-1 activity. To test our hypothesis, a nephropathy model of streptozotocin (STZ)-induced diabetes in rats was administered RSV with or without the HO-1 inhibitor ZnPP, and the effect of RSV on HO-1 expression and activity was investigated.

## 2. Materials and Methods

### 2.1. Drugs and Chemicals

Rosuvastatin calcium was purchased from Chemipharm Pharmaceutical Industry (6-October City, Egypt). ZnPP and STZ powders were obtained from Sigma-Aldrich Co. (St. Louis, MO, USA). Creatinine kit was purchased from HUMAN Gesellschaft für Biochemica und Diagnostica mbH (Wiesbaden, Germany). Kits for assessment of urea, reduced glutathione (GSH), and catalase were supplied by Biodiagnostic (Cairo, Egypt). Albumin, total cholesterol, and triglycerides kits were purchased from Spectrum (Cairo, Egypt). Antibodies against HO-1 and β-actin were purchased from Novus Biologicals (Littleton, CO, USA). Antibodies against TNF-α and caspase-3 were purchased from Thermo Fisher Scientific (Rockford, IL, USA). 

### 2.2. Animal Protocol and Experimental Design

Male Wistar rats weighing 200 ± 20 g were obtained from the Faculty of Agriculture, Animal Care Unit, Minia University. The council board of the Faculty of Pharmacy, Minia University, ethically approved the animal protocol (2017-0906). Rats were kept in regular laboratory animal housing cages, in a 12-h light/dark room, in a temperature of 24 °C ± 3. Standard laboratory animal chow (containing 20% proteins, 3% lipids and 12% fibers) and water were available ad libitum. After one week of acclimatization, animals were sub-divided into 5 groups of 10–14 animals each. The first group served as control. The second group received RSV for one month as a single daily dose of 10 mg/kg, freshly prepared in 0.5% carboxymethyl cellulose vehicle [14]. The other three groups were assigned for induction of DN using STZ as a single i.p. injection of 50 mg/kg body weight prepared in ice-cold 0.1 mol/L citrate buffer (pH 5) [15]. These groups were larger in number, as higher mortality was anticipated due to DN induction according to our preliminary pilots. After 3 days, diabetes was confirmed via assessment of blood glucose level, considering levels above 240 mg/dL as diabetic. A week after STZ injection, animals were left for a month to develop DN [9]. At the end of this month (considered 0-time), DN animals were assigned to various groups of treatments for another month, such that the third group was untreated and served as the diseased DN group, the fourth group was DN/RSV group receiving RSV as the second group, and the fifth group (DN/RSV/ZnPP) received both RSV as the second group, together with additional ZnPP (50 µmol/kg i.p. daily) dissolved in alkaline solution having 1–2 drops of 0.1N NaOH, titrated to pH 7.4 with 0.1 N HCl and then diluted with normal saline in the dark [16]. All rats’ weights were recorded at time of induction of diabetes and at the end of the experiment, where animals were fasted from food with free access to water for 12 h and urine samples were collected. After sacrifice via decapitation, blood samples were collected then centrifuged, and clear sera obtained were kept at −80 °C till used. Kidneys were quickly removed and weighed. Afterwards, longitudinal slices of the left kidney of each animal were taken for histopathological and Western blot examination. The right kidneys were snap-frozen in liquid nitrogen and stored at −80 °C until subsequently homogenized in cold potassium phosphate buffer (0.05 M, pH 7.4) for various biochemical analyses.

### 2.3. Biochemical Analysis

Using the OKmeter Direct Blood Glucose Monitoring Automatic System (Taiwan), glucose levels were assessed via blood samples from the tip of the rat tail. Biochemical analysis of serum and urinary creatinine and albumin levels were measured using commercially available kits. Similarly, commercial kits were used according to manufacturer’s instructions for assessment of serum urea and lipid profile including total cholesterol, triglycerides, low density lipoprotein-cholesterol (LDL-C) and high-density lipoprotein-cholesterol (HDL-C), as well as oxidative stress markers, as GSH and catalase. Serum and urinary Na^+^ and K^+^ levels were evaluated via flame photometer (Jenway PFP7). Determination of lipid peroxidation in the renal cortex in the form of thiobarbituric acid reacting substance was performed, expressed as equivalents of malondialdehyde (MDA), employing 1,1,3,3-tetramethoxypropane as standard [17]. The total level of renal tissue nitrite and nitrate was measured as a means for assessment of the renal nitric oxide (NO) level, by reduction of nitrate into nitrite using copperized cadmium, with subsequent color development using Griess reagent in acidic medium [18]. Superoxide dismutase (SOD) activity in renal tissue homogenate was determined according to the kit’s instructions, based on inhibition of SOD to phenazine methosulphate-induced reduction of nitroblue tetrazolium dye. Assessment of myeloperoxidase (MPO) activity in renal tissues was performed as described [19], where 1 unit of MPO was considered as the amount of MPO degrading 1 μm peroxide/min.

### 2.4. Renal Histopathology

Renal tissue slices were fixed in 10% neutral buffered formalin. After fixation, the tissue was dehydrated and embedded in paraffin, then sectioned and stained with hematoxylin and eosin (H&E) stain for histological examination via light microscopy (Olympus CX41). Blinded semi-quantitative microscopic analysis was performed, investigating slides from each animal, 3 fields/slide. Grading renal changes as mild, moderate, or severe (+, ++, or +++) was considered when fields showed as <25, 50 or 75% histopathological changes of total fields examined, respectively [20]. 

### 2.5. Western Blot Assay

Kidney tissue sections were homogenized in Tris-HCl lysis buffer (1 mL lysis buffer/100 mg tissue), at 3000 rpm on ice, then centrifuged for 20 min at 14,000 rpm at 4 °C. The supernatant was collected, and total protein concentration was evaluated [21]. Western blot was performed as previously described [22], with slight modifications. In brief, protein samples of 20 µg were separated via 10% sodium dodecyl sulfate-polyacrylamide gel electrophoresis (SDS-PAGE), then electrochemically transferred to nitrocellulose membrane for 1 h at 50 mA. Afterwards, membranes were submerged in Ponceau S to confirm protein blotting, then blocked with 5% skim milk in buffer of tris-saline-tween 20 (TBS-T). Membranes were incubated in 5% blocking solution overnight at 4 °C with primary antibodies against either HO-1 in ratio 1:1000, TNF-α in ratio 1:50, caspase 3 in ratio 1:50 and β-actin in ratio 1:1000 [23,24,25]. Membranes were washed three times, 10 min each using TBS-T at room temperature, and then incubated for 1 h at room temperature in blocking solution with secondary antibody (1:10,000 goat/anti-rabbit non-biotinylated alkaline phosphatase antibody). Membranes were washed again three times, 10 min each with TBS-T at room temperature. Bands were identified using enhanced chemiluminescence system. ImageJ program software was used for performing semi-quantitative determination of Western blotting products, such that relative expression of each of the tested proteins (R) was calculated by the following equation: R = densitometrical units of tested protein/densitometrical units of β-actin.

### 2.6. HO-1 Activity Assay Method

Renal tissue HO-1 activity was evaluated as previously described [26] and calculated by means of the product of heme degradation, bilirubin, which was extracted using chloroform, whose concentration was assessed using spectrophotometry. Homogenization of renal tissues was performed in 3 volumes of homogenization buffer and centrifuged at 3000 rpm for 4 min. To produce the mitochondrial pellet, another centrifugation was performed at 14,000 rpm for 5 min at 4 °C. The supernatant was collected, and the protein content was determined [21]. For measuring HO-1 activity, supernatant containing 10 mg of protein was added to a 100-µL HO-1 activity cocktail, composed of 96 mg of each of NADPH, NADH and glucose -6-phosphate, added to 1 mL MgCl2 (0.1 M) and completed to 10 mL with phosphate buffered saline (pH 7.4). Afterwards, 100 µL of G-6-PD, 10 µL of heme, 2 µL of MgCl2, 20 µL of KCl, and 50 µL of rat liver cytosol to provide biliverdin reductase were added and completed to 1 mL with phosphate buffer. Samples were incubated at 37 °C for 1 h in darkness and the reaction was terminated by cooling on ice. The bilirubin produced was extorted by adding 1 mL chloroform to each sample, mixed by a vortex for 30 s, centrifuged for 30 min at 3000 rpm and put in a −20 °C deep freezer wrapped in aluminum foil. The next day, samples were left to reach room temperature and the bilirubin produced in the samples was determined through a scanning spectrophotometer as the difference between the absorbance at 463 and 520 nm. Bilirubin values were calculated using a standard bilirubin curve. HO-1 activity was represented as pmol bilirubin/mg protein/1 h.

### 2.7. Statistical Analysis

Statistical calculation was performed using GraphPad Prism version 5.01 for Windows (GraphPad, San Diego, CA, USA). Results were represented as means ± S.E.M. Statistical significance was estimated using a one-way analysis of variance (ANOVA), with Tukey–Kramer as a post analysis test. Results were considered as having a statistically significant difference when p values are less than 0.05.

## 3. Results

### 3.1. The Effect on Weight and Blood Glucose Levels

In the current study, administration of RSV (10 mg/kg orally for 30 days) in non-diabetic rats did not affect either the change of body weight or kidney index (Table 1). Induction of DN, on the other hand, caused a significant decrease in change in body weight with a significant increase in kidney index. Administration of RSV in DN rats significantly reversed both effects compared to DN untreated group. Co-administration of ZnPP with RSV in DN rats significantly blocked the effect of RSV. Concerning serum glucose, the induction of diabetes via STZ significantly increased the serum glucose level compared to the control at both 0-time and after 60 days of induction, as compared to the control. Administration of either RSV alone or together with ZnPP in diabetic rats did not affect serum glucose levels compared to DN untreated group. 

### 3.2. The Effect on Biochemical Kidney Function Parameters and Serum Lipid Profile

Induction of DN was confirmed by a significant increase in serum levels of urea and creatinine, as well as serum cations, Na^+^ and K^+^, in addition to the urinary albumin-to-creatinine ratio, with a significant decrease in serum albumin as well as urinary Na^+^ and K^+^, as compared to non-diabetic control (Table 2). Administration of RSV in DN rats significantly reversed the effect of DN induction on all the previous parameters. On the other hand, co-administration of ZnPP blocked these effects of RSV, and retrieved levels not significant from the DN group.

Administrating RSV in non-diabetic rat had no effect on serum lipid profile compared to the control group (Table 3). Induction of DN caused a significant increase in serum total cholesterol, triglycerides, and LDL-C, with a significant decrease in serum HDL-C. The DN/RSV group, however, showed a significant decrease in serum total cholesterol, triglycerides, and LDL-C, with a significant increase in serum HDL-C, as compared to the DN group, which was reversed in the DN/RSV/ZnPP group.

### 3.3. The Effect on Renal Histopathology

**Figure 1:**
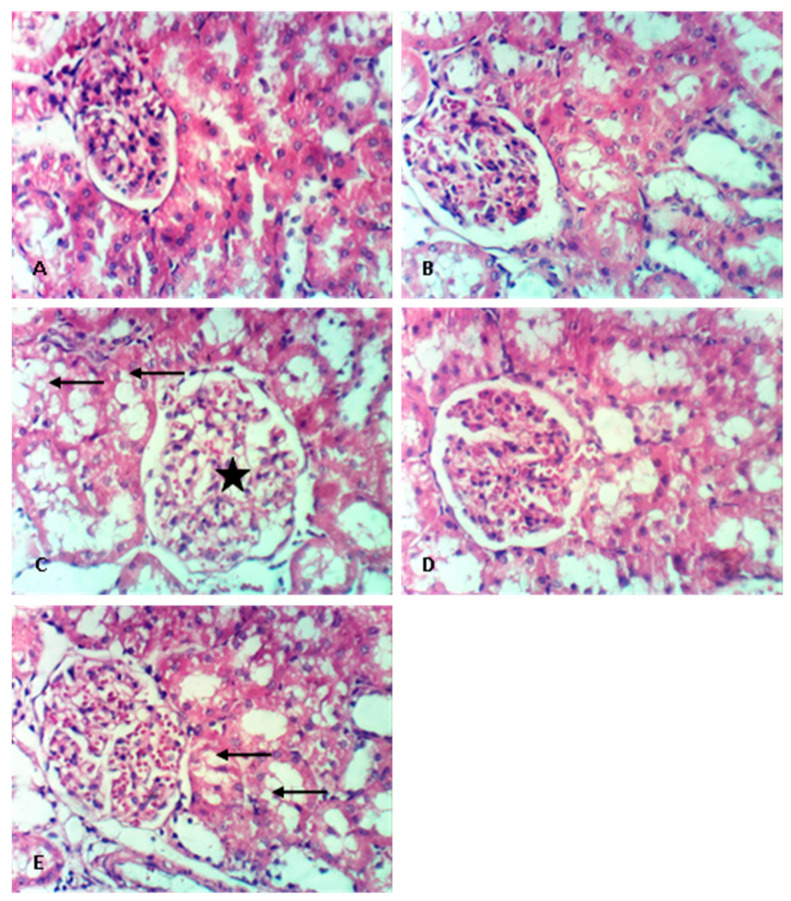
Effect of rosuvastatin (RSV) on renal histopathological changes in diabetic nephropathy (DN) in rats. Rat renal photomicrographs (400×) of (**A**) control, (**B**) RSV, (**C**) DN, (**D**) DN/RSV and (**E**) DN/RSV/zinc protoporphyrin-IX groups. Black arrow: vacuolation of epithelial lining renal tubules; star: distorted glomerulus.

Compared to control (Figure 1A), the renal histopathological picture was not affected by administration of RSV in non-diabetic rats (Figure 1B). To the contrary, the DN group showed a significant renal histopathological deterioration (Figure 1C), as scored in Table 4, having a marked degeneration of renal tubules that showed dilation and vacuolation of lining epithelium, in addition to substantial congestion of blood vessels, most remarkably at the glomerular tuft, permitting leukocytic infiltration. Administration of RSV in DN rats extensively improved renal histopathological pattern (Figure 1D), whereas co-administration of ZnPP with RSV in DN rats ameliorated the favorable effects of RSV (Figure 1E). Histopathological findings were scored, and results were summarized in Table 4.

### 3.4. The Effect on Renal Oxidative Stress Markers

In non-diabetic rats, RSV did not affect any of the renal oxidative stress markers tested (Table 5). Induction of DN caused a significant decrease in renal GSH level and the enzymatic activity of SOD and catalase, while significantly increasing renal levels of MDA, MPO and NO. Administration of RSV in DN rats caused a significantly opposite effect compared to DN untreated rats. Interestingly, co-administration of ZnPP and RSV in DN rats significantly returned oxidative stress markers to levels not significant from DN untreated rats.

### 3.5. The Effect on Renal HO-1 Expression and Activity

**Figure 2:**
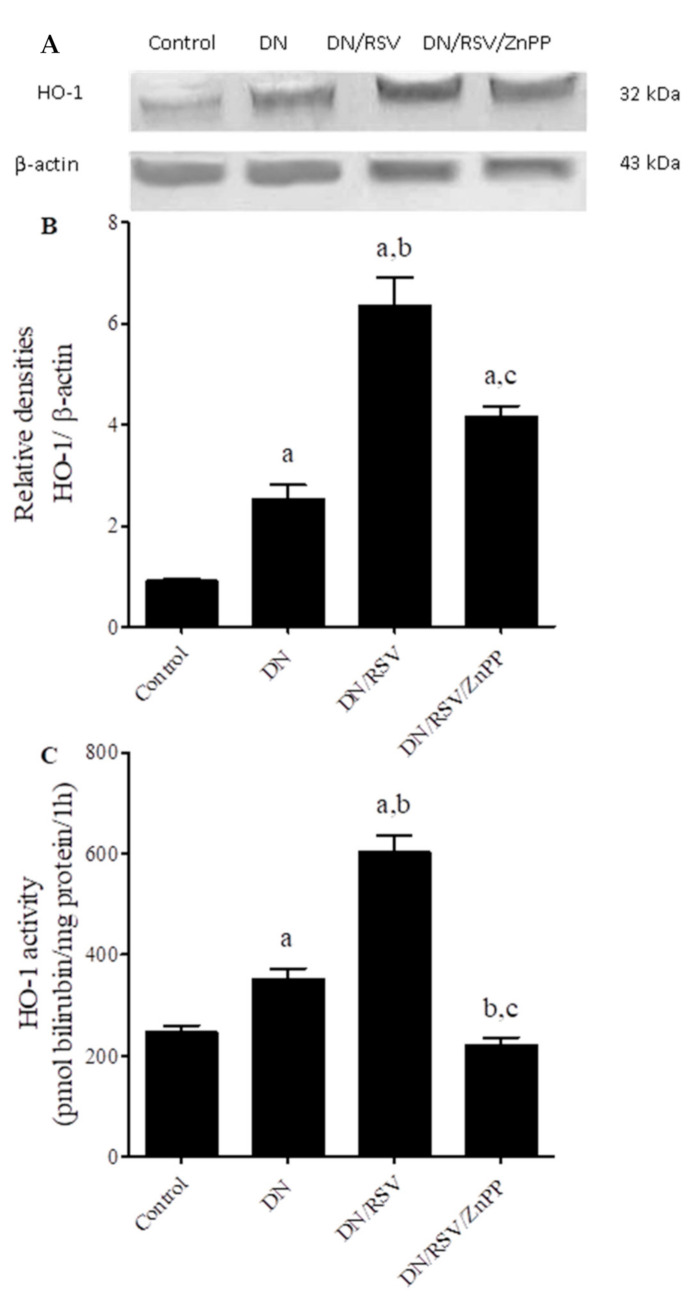
Effect of rosuvastatin (RSV) on renal Western blot analysis of heme oxygenase (HO)-1 expression and enzymatic activity in diabetic nephropathy (DN) in rats. ZnPP, zinc protoporphyrin-IX. (**A**) Representative Western blot showing HO-1 immune reactive protein and β-actin bands. Graph (**B**) shows relative densitometry of HO-1/β-actin ratio, where values are a representation of three blots as ratio means ± S.E.M. Graph (**C**) shows HO-1 enzymatic activity in different groups, where values are a representation of 6–11 observations as means ± S.E.M. Results are considered significantly different when *p* < 0.05. ^a^ Significant difference compared to control, ^b^ significant difference compared to DN group, ^c^ significant difference compared to DN/RSV group.

Using Western blot analysis, renal HO-1 showed higher expression in the DN group compared to the control (Figure 2A), which was confirmed with a significant difference in the relative densitometry of HO-1 to β-actin analysis of both groups (Figure 2B). Administration of RSV to DN rats further increased HO-1 expression to levels significantly higher than DN untreated rats. Co-administration of ZnPP with RSV in DN rats significantly decreased HO-1 renal expression and returned it to levels insignificant from those seen in DN untreated rats. Similarly, in the DN group, HO-1 enzymatic activity was significantly increased compared to the control, and even significantly further increased in the DN/RSV group compared to the DN group (Figure 2C). Co-administration of the HO-1 inhibitor ZnPP with RSV in DN rats decreased renal HO-1 activity to levels comparable to that of the non-diabetic control.

### 3.6. The Effect on Renal TNF-α and Caspase 3 Expression

**Figure 3:**
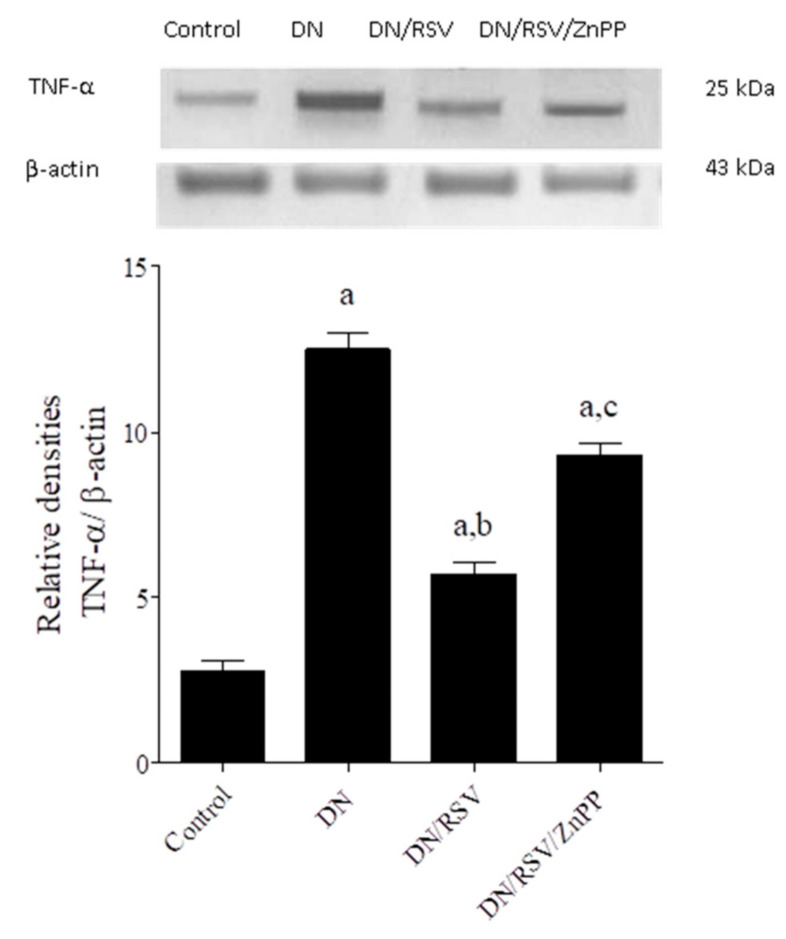
Effect of rosuvastatin (RSV) on renal Western blot analysis of tumor necrosis factor (TNF)-α expression in diabetic nephropathy (DN) in rats. ZnPP, zinc protoporphyrin-IX. Representative Western blot (**upper panel**) showing TNF-α immune reactive protein and β-actin bands. The graph shows relative densitometry of TNF-α/β-actin ratio (**lower panel**). Values are a representation of 3 blots as ratio means ± S.E.M and results are considered significantly different when *p* < 0.05. ^a^ Significant difference compared to control, ^b^ significant difference compared to DN group, ^c^ significant difference compared to DN/RSV group.

**Figure 4:**
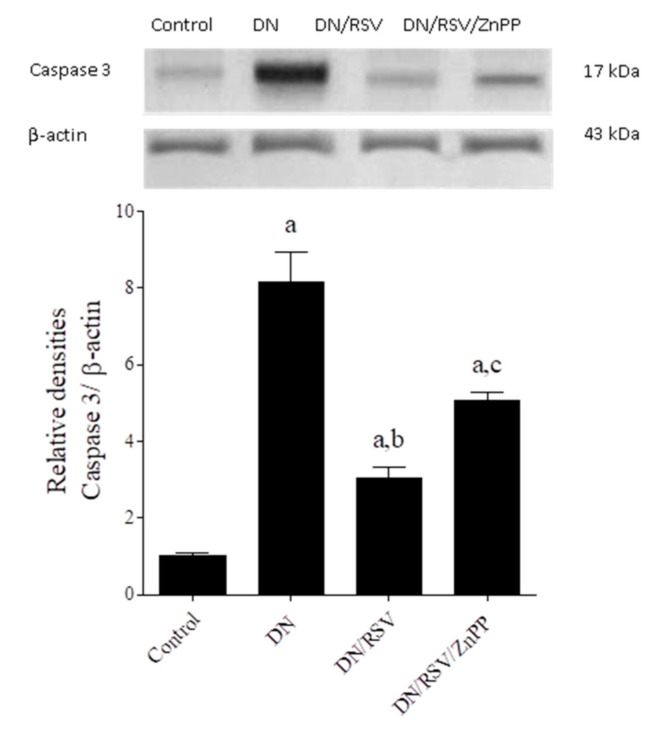
Effect of rosuvastatin (RSV) on renal Western blot analysis of caspase 3 expression in diabetic nephropathy (DN) in rats. ZnPP, zinc protoporphyrin-IX. Representative Western blot (**upper panel**) showing caspase 3 immune reactive protein and β-actin bands. Graph shows relative densitometry of caspase 3/β-actin ratio (**lower panel**). Values are a representation of three blots as ratio means ± S.E.M. Results are considered significantly different when *p* < 0.05. ^a^ Significant difference compared to control, ^b^ significant difference compared to DN group, ^c^ significant difference compared to DN/RSV group.

Renal expression of TNF-α and caspase 3 was assessed by Western blot and showed that the DN group had a significantly higher expression level of both proteins compared to the control (Figure 3 and Figure 4, respectively). Administration of RSV to DN rats caused a significant decrease in renal expression of both proteins compared to the DN group, while co-administration of ZnPP with RSV to DN rats caused a significant increase in the expression level of both proteins compared to the DN/RSV group.

## 4. Discussion

The patho-physiological causes of DN are comprised of a number of interrelated complex molecular pathways. Targeting these pathways may delay the progression, or even prevent the development of DN. In the current study, induction of diabetes, as expected, caused deterioration of kidney function and damaged its structure. Administering RSV succeeded in improving such damage. To date, the effect of RSV on the kidney was still debatable. In line with the current results, RSV nephroprotective effects have been reported against renal injury caused by contrast media [27,28], gentamicin [29], piroxicam [30], and high-cholesterol diet [31]. In DN, RSV has also shown potential beneficial nephroprotective effects by diminishing renal glomerular endothelial proliferation and suppressing interstitial fibrosis [32,33]. Furthermore, RSV has been reported to improve kidney function and ameliorate oxidative stress, independent of its lipid-modifying effects, in patients with DN [34]. To the contrary of all these beneficial effects, several studies indicated that RSV had harmful effects on the kidney. Administration of RSV was reported to cause proximal tubule damage and renal toxicity in rats [35] and acute interstitial nephritis in humans [36]. In addition, RSV caused renal damage associated with severe rhabdomyolysis, whether RSV was given alone [37,38,39], or in combination with colchicine [40], ticagrelor [41], or cocaine and heroin [42]. Despite that the results of the current study suggested that RSV had protective effects against DN, long-term follow-up studies are needed to confirm whether RSV improves DN or just delays its progress.

In the present study, induction of DN caused significant decreased activity of renal antioxidant enzymes and increased oxidative products, which was in line with previous studies documenting the role of oxidative stress in DN [4,13,43]. RSV succeeded in reversing the oxidative stress markers tested which is in line with previous animal studies [44], as well as human studies, reporting that RSV improved kidney function and reduced oxidative stress independent of its effect on lipid levels in patients with DN [34]. In the present study, the DN group also showed high expression of renal TNF-α, signifying the stimulation of inflammatory pathway and cytokine production, which was ameliorated by RSV administration. This is in line with the reported anti-inflammatory effect of RSV decreasing TNF-α in diabetic nephropathy [45], LPS-induced cardiac injury [46] and rheumatoid arthritis animal models [47]. Apoptosis in renal tissue, in the current study, was also seen after the induction of DN, as indicated by increased renal expression of the pro-apoptotic protein, caspase 3, which was reversed by RSV administration. It is hard to confirm whether such an effect was due to pure anti-apoptotic effect of RSV or indirect via inhibition of oxidative/inflammatory pathways, due to their complex crosstalk and intermingling interactions [5].

It was reported that HO-1 had a role in the prevention of DN, as HO-1 polymorphism was associated with type 2 diabetes mellitus in humans [48], and eventually DN, suggesting HO-1 as a target for therapy of acute renal injury [49]. We have also previously shown the role of HO-1 in protection against DN using hemin as an agonist and ZnPP as an antagonist of HO-1 enzymatic activity [9]. Here, we confirm that the induction of DN caused a mild increase in renal HO-1 activity and protein expression, probably as a feedback mechanism to protect the kidney against DN hazards. Our findings were in line with several previous studies reporting similar increase in HO-1 activity and expression in diabetic animal models [6,50,51,52,53]. One study reported, in contradiction with our results, that diabetic high-fat-diet/STZ-treated mice kidneys showed a decrease in HO-1 expression [54]. This is probably due to the different animal species used, with different DN induction methods using a high-fat diet for 22 weeks together with low doses of STZ. 

Here, the present study shows that RSV has hemin-like stimulatory effect on renal HO-1 expression and/or activity in DN rats. At the expression level, RSV was previously reported to induce HO-1 expression in vitro in neuro-2A cells exposed to lipopolysaccharide [55], cultured HL-1 cell line of atrium-derived myocytes [56], in aortic tissue of AngII-ApoE-/- murine animal model [57], umbilical vein endothelial cells [58] and myocardial hypertrophy in rats [59]. Concerning the effect on HO-1 activity, RSV was also reported to have stimulatory effect on HO-1 activity on different other tissues as the lungs [60]. However, this is not indicative of how RSV may affect HO-1 in the kidney, as RSV was reported to have differential effect on HO-1 activity in various tissues, where it increased HO-1 activity in heart > lung > brain, with no effect on the liver [61], indicating that RSV effect on HO-1 activity is organ-specific.

Interestingly, administration of the HO-1 inhibitor, ZnPP, concomitantly with RSV, reversed the beneficial effects of RSV on oxidative stress, inflammatory and pro-apoptotic parameters tested. These findings suggest that RSV-mediated nephroprotective effects are governed by its hemin-like effect on HO-1. Indeed, HO-1 enzyme has a pivotal role in cellular defense mechanisms against oxidative stress, by degrading heme to strong antioxidants including biliverdin, carbon monoxide and free iron [62], which might explain why, in the current study, the anti-oxidative effects of RSV were abolished by concomitant administration of ZnPP; an inhibitor of HO-1. Nevertheless, the anti-inflammatory effect of RSV was also significantly reduced by co-administration of ZnPP in the present study. It is possible that the anti-inflammatory effect of RSV is mediated indirectly through inhibition of the production of triggering ROS due HO-1 antioxidant effects. Nevertheless, a direct linkage between HO-1 activity and the inflammatory pathway in kidney diseases has been previously suggested in cisplatin-induced nephrotoxicity [63,64]. In addition, peritoneal macrophages from HO-1+/− mice were reported to have increased gene expression of pro-inflammatory cytokines, including TNF-α [57]. Furthermore, we have recently shown that direct stimulatory effect of HO-1 activity by hemin can inhibit renal expression of TNF-α in vivo [9]. The anti-apoptotic effect of RSV was also ameliorated in the present study by concomitant administration of ZnPP. This finding is in accordance with previous studies suggesting that, under diabetic conditions, inhibition of HO-1 magnified the amount of renal apoptotic cells both in vivo and in vitro [65]. It is difficult, though, to suggest from the results of the current study a cause–effect relationship between oxidative, inflammatory, and apoptotic pathways in DN, as ROS may initiate abnormal downstream signaling molecules, causing further activation of both inflammatory and apoptotic signals [43], as the induction of TNF-α or pro-apoptotic caspases, respectively. However, inflammation and/or apoptosis processes might themselves increase ROS production [5]. Still, since inhibiting RSV-induced HO-1 by ZnPP in the current study blocked the beneficial effects of RSV on all three pathways, it is logical to assume that HO-1 is upstream to all of them, whatever complex their cause–effect relation is. It is also hard to suggest from the present study whether the effect of increased renal HO-1 activity seen due to administration of RSV in DN rats was due to pure agonistic effect on HO-1 enzymatic function, or due to RSV induction of HO-1 renal protein expression that, in turn, would increase the activity, especially that co-administration of ZnPP inhibited the effect of RSV on both parameters. Yet, it is noteworthy that the inhibitory effect of ZnPP on enzymatic functional activity in RSV-treated DN rats was more profound than the inhibition at renal HO-1 protein expression level. 

## 5. Conclusions

The beneficial effect of RSV in DN is most probably attributed to RSV-mediated antioxidant, anti-inflammatory and/or anti-apoptotic properties. These beneficial properties of RSV can, at least in part, be blocked by co-administration of ZnPP, which is a HO-1 inhibitor, suggesting a definite crosstalk between renal HO-1 enzyme and these pathways contributing to the initiation and progress of DN. Further mechanistic investigations are necessary to elucidate whether the effect of RSV is purely agonistic on HO-1 enzymatic activity or is mainly due to regulatory effect on HO-1 genetic expression.

## Figures and Tables

**Table 1:** Effect of rosuvastatin (RSV) on change in body weight, kidney index and blood glucose in diabetic nephropathy (DN) in rats.

	% ∆ Body Weight	Kidney Index	Blood Glucose at 0-Time (mg/dL)	Blood Glucose at Day 60 (mg/dL)
Control	134 ± 3	0.52 ± 0.02	126 ± 3	148 ± 6
RSV	135 ± 2	0.5 ± 0.04	128 ± 8	112 ± 10
DN	85 ± 1 ^a^	1.0 ± 0.05 ^a^	542 ± 63 ^a^	464 ± 70 ^a^
DN/RSV	103 ± 1 ^a,b^	0.59 ± 0.03 ^b^	504 ± 41 ^a^	462 ± 49 ^a^
DN/RSV/ZnPP	88 ± 2 ^a,c^	0.85 ± 0.05 ^a,c^	458 ± 79 ^a^	538 ± 29 ^a^

Estimation of percent of change (% ∆) in body weight by the equation = 100 × (final/initial body weight). ZnPP, zinc protoporphyrin-IX. Values are a representation of 6–11 observations as means ± S.E.M. When *p* is less than 0.05, results are reported to be significantly different. ^a^ Significant difference compared to control, ^b^ significant difference compared to DN group, ^c^ significant difference compared to DN/RSV group.

**Table 2:** Effect of rosuvastatin (RSV) on kidney functional parameters in diabetic nephropathy (DN) in rats.

	Control	RSV	DN	DN/RSV	DN/RSV/ZnPP
Serum Urea (mg/dL)	11 ± 1	10 ± 1	24 ± 1 ^a^	12 ± 2 ^b^	28 ± 1 ^a,c^
Serum Creatinine (mg/dL)	0.89 ± 0.10	0.98 ± 0.08	4.00 ± 0.13 ^a^	1.2 ± 0.06 ^b^	3.6 ± 0.29 ^a,c^
Serum Albumin (g/dL)	3.4 ± 0.3	3.1 ± 0.2	2.0 ± 0.1 ^a^	3.2 ± 0.2 ^b^	2.2 ± 0.1 ^a,c^
ACR (mg/g)	17 ± 1	13 ± 1	193 ± 23 ^a^	69 ± 10 ^b^	187 ± 18 ^a,c^
Serum Na^+^ (mEq/L)	134 ± 2	133 ± 12	210 ± 5 ^a^	145 ± 6 ^b^	230 ± 18 ^a,c^
Urine Na^+^ (mEq/L)	937 ± 24	1063 ± 46	393 ± 23 ^a^	863 ± 75 ^b^	532 ± 6 ^a,c^
Serum K^+^ (mEq/L)	3.4 ± 0.2	4.1 ± 0.2	7.6 ± 0.5 ^a^	4.6 ± 0.3 ^b^	6.3 ± 0.3 ^a^
Urine K^+^ (mEq/L)	89 ± 6	83 ± 5	22 ± 1 ^a^	44 ± 5 ^a,b^	27.8 ± 1.9 ^a^

ACR, urinary albumin/creatinine ratio; ZnPP, zinc protoporphyrin-IX. Values are a representation of 6-11 observations as means ± S.E.M. When *p* is less than 0.05, results are reported to be significantly different. ^a^ Significant difference compared to control, ^b^ significant difference compared to DN group, ^c^ significant difference compared to DN/RSV group.

**Table 3:** Effect of rosuvastatin (RSV) on lipid profile in diabetic nephropathy (DN) in rats.

	Total Cholesterol (mg/dL)	Triglycerides (mg/dL)	HDL-C (mg/dL)	LDL-C (mg/dL)
Control	70 ± 1	36.6 ± 0.5	50 ± 3	14 ± 2
RSV	70 ± 3	36.0 ± 0.3	51 ± 3	14 ± 1
DN	79 ± 2 ^a^	49.3 ± 1.9 ^a^	22 ± 3 ^a^	45 ± 2 ^a^
DN/RSV	63 ± 2 ^b^	36.1 ± 1.6 ^b^	50 ± 2 ^b^	23 ± 2 ^b^
DN/RSV/ZnPP	64 ± 2 ^b^	38.9 ± 1.7 ^b^	45 ± 4 ^b^	22 ± 2 ^b^

ZnPP, zinc protoporphyrin-IX. Values are a representation of 6–11 observations as means ± S.E.M. When *p* is less than 0.05, results are reported to be significantly different. ^a^ Significant difference compared to control, ^b^ significant difference compared to DN group.

**Table 4:** Effect of rosuvastatin (RSV) on renal histopathological picture scoring in diabetic nephropathy (DN) in rats.

	Tubular Degeneration	Tubular Dilation	Congested Blood Vessels	Leukocytic Infiltration	Vacuolation of Lining Epithelium	Congestion of Glomerular Tuft
Control	-	-	-	-	-	-
RSV	-	-	-	-	-	-
DN	++	++	+++	+++	+++	+++
DN/RSV	-	+	+	+	+	+
DN/RSV/ZNPP	++	+	++	+++	++	++

ZnPP, zinc protoporphyrin-IX. Normal histological feature was indicated by (-). Levels +, ++ and +++ were considered mild, moderate and severe degrees of less than 25, 50 and 75% histopathological distortion in total fields tested, respectively. Values are of renal tissues from each section of each animal, 3 fields/section (400×).

**Table 5:** Effect of rosuvastatin (RSV) on renal tissue oxidative stress markers in diabetic nephropathy (DN) in rats.

	GSH (mmol/g Tissue)	SOD (U/g Tissue)	Catalase (U/g Tissue)	MDA (nmol/g Tissue)	MPO (U/g Tissue)	NO (nmol/g Tissue)
Control	56 ± 3	1199 ± 62	433 ± 11	200 ± 3	0.12 ± 0.02	112 ± 3
RSV	50 ± 4	1138 ± 80	397 ± 14	189 ± 4	0.11 ± 0.02	124 ± 10
DN	29 ± 2 ^a^	629 ± 70 ^a^	135 ± 19 ^a^	352 ± 8 ^a^	0.32 ± 0.04 ^a^	264 ± 8 ^a^
DN/RSV	48 ± 2 ^b^	1131 ± 73 ^b^	320 ± 21 ^a,b^	305 ± 10 ^a,b^	0.17 ± 0.01 ^b^	131 ± 9 ^b^
DN/RSV/ZNPP	32 ± 2 ^a,c^	451 ± 52 ^a,c^	198 ± 20 ^a,c^	361 ± 8 ^a,c^	0.30 ± 0.04 ^a,c^	248 ± 24 ^a,c^

GSH, reduced glutathione; SOD, superoxide dismutase; MDA, malondialdehyde; MPO, myeloperoxidase; NO, nitric oxide; ZnPP, zinc protoporphyrin-IX. Values are a representation of 6–11 observations as means ± S.E.M. When *p* is less than 0.05, results are reported to be significantly different. ^a^ Significant difference compared to control, ^b^ significant difference compared to DN group, ^c^ significant difference compared to DN/RSV group.

## References

[1] Mohsen M., Elberry A.A., Mohamed R.A., Abdelrahim M.E.A., Hussein R.R.S. (2021). Recent therapeutic targets in diabetic nephropathy. Int. J. Clin. Pract..

[2] Maiti A.K. (2021). Development of Biomarkers and Molecular Therapy Based on Inflammatory Genes in Diabetic Nephropathy. Int. J. Mol. Sci..

[3] Schiffer T.A., Friederich-Persson M. (2017). Mitochondrial Reactive Oxygen Species and Kidney Hypoxia in the Development of Diabetic Nephropathy. Front. Physiol..

[4] Fakhruddin S., Alanazi W., Jackson K.E. (2017). Diabetes-Induced Reactive Oxygen Species: Mechanism of Their Generation and Role in Renal Injury. J. Diabetes Res..

[5] Taye A., El-Sheikh A.A. (2013). Lectin-like oxidized low-density lipoprotein receptor 1 pathways. Eur. J. Clin. Investig..

[6] Yuan S., Liang X., He W., Liang M., Jin J., He Q. (2021). ATF4-dependent heme-oxygenase-1 attenuates diabetic nephropathy by inducing autophagy and inhibiting apoptosis in podocyte. Ren. Fail..

[7] Tesch G.H. (2017). Diabetic nephropathy—Is this an immune disorder?. Clin. Sci..

[8] Li Z., Xu Y., Liu X., Nie Y., Zhao Z. (2017). Urinary heme oxygenase-1 as a potential biomarker for early diabetic nephropathy. Nephrology.

[9] Ali M.A.M., Heeba G.H., El-Sheikh A.A.K. (2017). Modulation of heme oxygenase-1 expression and activity affects streptozotocin-induced diabetic nephropathy in rats. Fundam. Clin. Pharmacol..

[10] Chen Y., Zhi Y., Li C., Liu Y., Zhang L., Wang Y., Che K. (2016). HDL cholesterol and risk of diabetic nephropathy in patient with type 1 diabetes: A meta-analysis of cohort studies. Diabetes Res. Clin. Pract..

[11] Cortese F., Gesualdo M., Cortese A., Carbonara S., Devito F., Zito A., Ricci G., Scicchitano P., Ciccone M.M. (2016). Rosuvastatin: Beyond the cholesterol-lowering effect. Pharmacol. Res..

[12] Palaniswamy C., Selvaraj D.R., Selvaraj T., Sukhija R. (2010). Mechanisms underlying pleiotropic effects of statins. Am. J. Ther..

[13] Sharma D., Bhattacharya P., Kalia K., Tiwari V. (2017). Diabetic nephropathy: New insights into established therapeutic paradigms and novel molecular targets. Diabetes Res. Clin. Pract..

[14] Heeba G.H., Hamza A.A. (2015). Rosuvastatin ameliorates diabetes-induced reproductive damage via suppression of oxidative stress, inflammatory and apoptotic pathways in male rats. Life Sci..

[15] Vaishya R., Singh J., Lal H. (2008). Effect of irbesartan on streptozotocin induced diabetic nephropathy: An interventionary study. Indian J. Clin. Biochem..

[16] Wen T., Wu Z.M., Liu Y., Tan Y.F., Ren F., Wu H. (2007). Upregulation of heme oxygenase-1 with hemin prevents D-galactosamine and lipopolysaccharide-induced acute hepatic injury in rats. Toxicology.

[17] Buege J.A., Aust S.D. (1978). Microsomal lipid peroxidation. Methods Enzymol..

[18] Sastry K.V., Moudgal R.P., Mohan J., Tyagi J.S., Rao G.S. (2002). Spectrophotometric determination of serum nitrite and nitrate by copper-cadmium alloy. Anal. Biochem..

[19] Hillegass L.M., Griswold D.E., Brickson B., Albrightson-Winslow C. (1990). Assessment of myeloperoxidase activity in whole rat kidney. J. Pharmacol. Methods.

[20] El-Sheikh A.A., Morsy M.A., Al-Taher A.Y. (2016). Protective mechanisms of resveratrol against methotrexate-induced renal damage may involve BCRP/ABCG2. Fundam. Clin. Pharmacol..

[21] Lowry O.H., Rosebrough N.J., Farr A.L., Randall R.J. (1951). Protein measurement with the Folin phenol reagent. J. Biol. Chem..

[22] Laemmli U.K. (1970). Cleavage of structural proteins during the assembly of the head of bacteriophage T4. Nature.

[23] Heeba G.H., Hamza A.A., Hassanin S.O. (2016). Induction of heme oxygenase-1 with hemin alleviates cisplatin-induced reproductive toxicity in male rats and enhances its cytotoxicity in prostate cancer cell line. Toxicol. Lett..

[24] Zolotnik I.A., Figueroa T.Y., Yaspelkis B.B. (2012). Insulin receptor and IRS-1 co-immunoprecipitation with SOCS-3, and IKKalpha/beta phosphorylation are increased in obese Zucker rat skeletal muscle. Life Sci..

[25] Gezginci-Oktayoglu S., Tunali S., Yanardag R., Bolkent S. (2008). Effects of Z-FA.FMK on D-galactosamine/tumor necrosis factor-alpha-induced kidney injury and oxidative stress in mice: Effects of Z-FA.FMK on TNF-alpha-mediated kidney injury. Mol. Cell Biochem..

[26] Abraham N.G., Lutton J.D., Levere R.D. (1985). Heme metabolism and erythropoiesis in abnormal iron states: Role of delta-aminolevulinic acid synthase and heme oxygenase. Exp. Hematol..

[27] Jiang Z., Zhang J., Lu Y. (2020). Protective Effects and Mechanisms of Rosuvastatin on Acute Kidney Injury Induced by Contrast Media in Rats. Int. J. Nephrol..

[28] Lin M., Xu T., Zhang W., Li D., Li Y., Hong X., Luan Y., Zhang W., Wang M. (2021). Effect of statins on post-contrast acute kidney injury: A multicenter retrospective observational study. Lipids Health Dis..

[29] Rasheed H.A., Al-Kuraishy H.M., Al-Gareeb A.I. (2019). Rosuvastatin Attenuates acute nephrotoxicity through modulation of oxidative stress in Sprague Dawley rats. J. Pak. Med. Assoc..

[30] Abdeen A., Aboubakr M., Elgazzar D., Abdo M., Abdelkader A., Ibrahim S., Elkomy A. (2019). Rosuvastatin attenuates piroxicam-mediated gastric ulceration and hepato-renal toxicity in rats. Biomed. Pharmacother..

[31] Jabarpour M., Rashtchizadeh N., Ghorbani H.A., Argani H., Nemati M., Dastmalchi S., Roshangar L., Ranjbarzadhag M., Mesgari-Abbasi M., Bargahi N. (2020). Protection of renal damage by HMG-CoA inhibitors: A comparative study between atorvastatin and rosuvastatin. Iran J. Basic Med. Sci..

[32] Kim D.H., Choi B.H., Ku S.K., Park J.H., Oh E., Kwak M.K. (2016). Beneficial Effects of Sarpogrelate and Rosuvastatin in High Fat Diet/Streptozotocin-Induced Nephropathy in Mice. PLoS ONE.

[33] Deng J., Wu G., Yang C., Li Y., Jing Q., Han Y. (2015). Rosuvastatin attenuates contrast-induced nephropathy through modulation of nitric oxide, inflammatory responses, oxidative stress and apoptosis in diabetic male rats. J. Transl. Med..

[34] Abe M., Maruyama N., Okada K., Matsumoto S., Matsumoto K., Soma M. (2011). Effects of lipid-lowering therapy with rosuvastatin on kidney function and oxidative stress in patients with diabetic nephropathy. J. Atheroscler. Thromb..

[35] Dodiya H., Jain M., Goswami S.S. (2011). Renal toxicity of lisinopril and rosuvastatin, alone and in combination, in Wistar rats. Int. J. Toxicol..

[36] Annigeri R.A., Mani R.M. (2015). Acute interstitial nephritis due to statin and its class effect. Indian J. Nephrol..

[37] Vijayakanthi N., Felner E.I., Romero R., Daley T.C. (2021). Rhabdomyolysis due to rosuvastatin in a patient with ROHHAD syndrome. J. Clin. Lipidol..

[38] Nikalji R., Sen S. (2021). Rosuvastatin-Induced Rhabdomyolysis: A Case Report. Indian J. Nephrol..

[39] Petreski T., Piko N., Petrijan T., Dvoršak B., Hojs R., Bevc S. (2021). Statin-Associated Necrotizing Myopathy Leading to Acute Kidney Injury: A Case Report. Case Rep. Nephrol. Dial..

[40] Sabanis N., Paschou E., Drylli A., Papanikolaou P., Zagkotsis G. (2021). Rosuvastatin and Colchicine combined myotoxicity: Lessons to be learnt. CEN Case Rep..

[41] Sibley R.A., Katz A., Papadopoulos J. (2021). The Interaction between Rosuvastatin and Ticagrelor Leading to Rhabdomyolysis: A Case Report and Narrative Review. Hosp. Pharm..

[42] Mitaritonno M., Lupo M., Greco I., Mazza A., Cervellin G. (2021). Severe rhabdomyolysis induced by co-administration of cocaine and heroin in a 45 years old man treated with rosuvastatin: A case report. Acta Biomed..

[43] Bhattacharjee N., Barma S., Konwar N., Dewanjee S., Manna P. (2016). Mechanistic insight of diabetic nephropathy and its pharmacotherapeutic targets: An update. Eur. J. Pharmacol..

[44] Hussein M.M., Mahfouz M.K. (2016). Effect of resveratrol and rosuvastatin on experimental diabetic nephropathy in rats. Biomed. Pharmacother..

[45] El-Sawaf E.S., Saleh S., Abdallah D.M., Ahmed K.A., El-Abhar H.S. (2021). Vitamin D and rosuvastatin obliterate peripheral neuropathy in a type-2 diabetes model through modulating Notch1, Wnt-10α, TGF-β and NRF-1 crosstalk. Life Sci..

[46] Ren G., Zhou Q., Lu M., Wang H. (2021). Rosuvastatin corrects oxidative stress and inflammation induced by LPS to attenuate cardiac injury by inhibiting the NLRP3/TLR4 pathway. Can. J. Physiol. Pharmacol..

[47] Qasim S., Alamgeer K.S., Shahzad M., Bukhari I.A., Vohra F., Afzal S. (2021). Rosuvastatin Attenuates Rheumatoid Arthritis-Associated Manifestations via Modulation of the Pro- and Anti-inflammatory Cytokine Network: A Combination of In Vitro and In Vivo Studies. ACS Omega.

[48] Bao W., Song F., Li X., Rong S., Yang W., Wang D., Xu J., Fu J., Zhao Y., Liu L. (2010). Association between heme oxygenase-1 gene promoter polymorphisms and type 2 diabetes mellitus: A HuGE review and meta-analysis. Am. J. Epidemiol..

[49] Bolisetty S., Zarjou A., Agarwal A. (2017). Heme Oxygenase 1 as a Therapeutic Target in Acute Kidney Injury. Am. J. Kidney Dis..

[50] Shen X., Hu B., Xu G., Chen F., Ma R., Zhang N., Liu J., Ma X., Zhu J., Wu Y. (2017). Activation of Nrf2/HO-1 Pathway by Glycogen Synthase Kinase-3beta Inhibition Attenuates Renal Ischemia/Reperfusion Injury in Diabetic Rats. Kidney Blood Press Res..

[51] Wen Y., Liu Y., Huang Q., Liu R., Liu J., Zhang F., Liu S., Jiang Y. (2021). Moringa oleifera Lam. seed extract protects kidney function in rats with diabetic nephropathy by increasing GSK-3β activity and activating the Nrf2/HO-1 pathway. Phytomedicine.

[52] Yang G., Li Q., Peng J., Jin L., Zhu X., Zheng D., Zhang Y., Wang R., Song Y., Hu W. (2021). Fucoxanthin regulates Nrf2 signaling to decrease oxidative stress and improves renal fibrosis depending on Sirt1 in HG-induced GMCs and STZ-induced diabetic rats. Eur. J. Pharmacol..

[53] Chen N., Song S., Yang Z., Wu M., Mu L., Zhou T., Shi Y. (2021). ChREBP deficiency alleviates apoptosis by inhibiting TXNIP/oxidative stress in diabetic nephropathy. J. Diabetes Complicat..

[54] Park J.H., Choi B.H., Ku S.K., Kim D.H., Jung K.A., Oh E., Kwak M.K. (2017). Amelioration of high fat diet-induced nephropathy by cilostazol and rosuvastatin. Arch. Pharm. Res..

[55] Hsieh C.H., Jeng J.C., Hsieh M.W., Chen Y.C., Lu T.H., Rau C.S., Jeng S.F. (2011). Involvement of the p38 pathway in the differential induction of heme oxygenase-1 by statins in Neuro-2A cells exposed to lipopolysaccharide. Drug Chem. Toxicol..

[56] Yeh Y.H., Kuo C.T., Chang G.J., Chen Y.H., Lai Y.J., Cheng M.L., Chen W.J. (2015). Rosuvastatin suppresses atrial tachycardia-induced cellular remodeling via Akt/Nrf2/heme oxygenase-1 pathway. J. Mol. Cell Cardiol..

[57] Azuma J., Wong R.J., Morisawa T., Hsu M., Maegdefessel L., Zhao H., Kalish F., Kayama Y., Wallenstein M.B., Deng A.C. (2016). Heme Oxygenase-1 Expression Affects Murine Abdominal Aortic Aneurysm Progression. PLoS ONE.

[58] Brownfoot F.C., Tong S., Hannan N.J., Hastie R., Cannon P., Kaitu’u-Lino T.J. (2016). Effects of simvastatin, rosuvastatin and pravastatin on soluble fms-like tyrosine kinase 1 (sFlt-1) and soluble endoglin (sENG) secretion from human umbilical vein endothelial cells, primary trophoblast cells and placenta. BMC Pregnancy Childbirth.

[59] Wang P., Luo L., Shen Q., Shi G., Mohammed A., Ni S., Wu X. (2018). Rosuvastatin improves myocardial hypertrophy after hemodynamic pressure overload via regulating the crosstalk of Nrf2/ARE and TGF-β/smads pathways in rat heart. Eur. J. Pharmacol..

[60] Dolkart O., Amar E., Shapira S., Marmor S., Steinberg E.L., Weinbroum A.A. (2015). Protective effects of rosuvastatin in a rat model of lung contusion: Stimulation of the cyclooxygenase 2-prostaglandin E-2 pathway. Surgery.

[61] Hsu M., Muchova L., Morioka I., Wong R.J., Schröder H., Stevenson D.K. (2006). Tissue-specific effects of statins on the expression of heme oxygenase-1 in vivo. Biochem. Biophys. Res. Commun..

[62] Negi G., Nakkina V., Kamble P., Sharma S.S. (2015). Heme oxygenase-1, a novel target for the treatment of diabetic complications: Focus on diabetic peripheral neuropathy. Pharmacol. Res..

[63] Ansari M.A. (2017). Sinapic acid modulates Nrf2/HO-1 signaling pathway in cisplatin-induced nephrotoxicity in rats. Biomed. Pharmacother..

[64] Potočnjak I., Broznić D., Kindl M., Kropek M., Vladimir-Knežević S., Domitrović R. (2017). Stevia and stevioside protect against cisplatin nephrotoxicity through inhibition of ERK1/2, STAT3, and NF-kappaB activation. Food Chem. Toxicol..

[65] Lee S.C., Han S.H., Li J.J., Lee S.H., Jung D.S., Kwak S.J., Kim S.H., Kim D.K., Yoo T.H., Kim J.H. (2009). Induction of heme oxygenase-1 protects against podocyte apoptosis under diabetic conditions. Kidney Int..

